# Muscle MRI reveals distinct abnormalities in genetically proven non-dystrophic myotonias^☆^

**DOI:** 10.1016/j.nmd.2013.05.001

**Published:** 2013-08

**Authors:** Jasper M. Morrow, Emma Matthews, Dipa L. Raja Rayan, Arne Fischmann, Christopher D.J. Sinclair, Mary M. Reilly, John S. Thornton, Michael G. Hanna, Tarek A. Yousry

**Affiliations:** a MRC Centre for Neuromuscular Diseases, Department of Molecular Neurosciences, UCL Institute of Neurology, London, UK; b University of Basel, Department of Radiology, Basel, Switzerland; c Academic Neuroradiological Unit, Department of Brain Repair and Rehabilitation, UCL Institute of Neurology, London, UK

**Keywords:** Non-dystrophic myotonia, MRI, Myotonia congenita, Paramyotonia congenita

## Abstract

We assessed the presence, frequency and pattern of MRI abnormalities in non-dystrophic myotonia patients. We reviewed T1-weighted and STIR (short-tau-inversion-recovery) 3T MRI sequences of lower limb muscles at thigh and calf level in 21 patients with genetically confirmed non-dystrophic myotonia: 11 with CLCN1 mutations and 10 with SCN4A mutations, and 19 healthy volunteers. The MRI examinations of all patients showed hyperintensity within muscles on either T1-weighted or STIR images. Mild extensive or marked T1-weighted changes were noted in 10/21 patients and no volunteers. Muscles in the thigh were equally likely to be affected but in the calf there was sparing of tibialis posterior. Oedema was common in calf musculature especially in the medial gastrocnemius with STIR hyperintensity observed in 18/21 patients. In 10/11 CLCN1 patients this included a previously unreported “central stripe”, also present in 3/10 SCN4A patients but no volunteers. Degree of fatty infiltration correlated with age (rho = 0.46, *p* < 0.05). Muscle MRI is frequently abnormal in non-dystrophic myotonia providing evidence of fatty infiltration and/or oedema. The pattern is distinct from other myotonic disorders; in particular the “central stripe” has not been reported in other conditions. Correlations with clinical parameters suggest a potential role for MRI as a biomarker.

## 1 Introduction

In many neuromuscular disorders MRI plays an important role in diagnosis and an increasing role in disease monitoring [1,2]. However there is little published on the MRI findings in patients with non-dystrophic myotonia (NDM). NDMs are the commonest skeletal muscle channelopathy and are classified as myotonia congenita (MC), paramyotonia congenita (PMC) or sodium channel myotonia (SCM) according to their clinical features [3]. Both dominant and recessive forms of myotonia congenita are caused by mutations in the CLCN1 gene [4] whilst both PMC and SCM are caused by mutations in the SCN4A gene [5]. Each subgroup of NDM has a distinct clinical phenotype but there can be overlap between the groups and also with myotonic dystrophy type 2. Advanced electrophysiological techniques aid genetic testing [6], but a significant minority may need analysis of several candidate genes before a diagnosis is reached.

MRI has an established role in the diagnostic work-up of many neuromuscular disorders [1,2]. Typical findings are areas of hyperintensity within muscles on T1-weighted (T1w) images due to fatty infiltration [7]. The pattern of fatty infiltration can help determine the defective gene responsible [8], particularly in congenital myopathies [1]. Hyperintensity on fat-suppressed T2-weighted sequences such as short-tau-inversion-recovery (STIR) represent muscle oedema which may be due to toxic, metabolic or inflammatory changes [9]. In skeletal muscle channelopathies published MRI research is more limited. Whilst significant T1 and STIR abnormalities have been reported in hypokalemic periodic paralysis [10], few [11] or no [12] abnormalities have been reported in NDM using T1w or STIR MRI sequences.

The objective of this study was to define the frequency and nature of any abnormalities on lower limb MRI in patients with genetically proven NDM compared with healthy volunteers.

## 2 Materials and methods

### 2.1 Subjects

In a 12 month period between June 2009 and June 2010 we identified 21 patients (12 males and 9 females, mean age 45.6 years, standard deviation 14.4 years, range 19–68 years) with genetically confirmed non-dystrophic myotonia seen at our service in whom MRI had been undertaken using the imaging protocol detailed below. The local ethics committee confirmed that the project conformed to research governance arrangements. The NDM patients included the following subtypes: dominant MC (*n* = 3), recessive MC (*n* = 8), PMC (*n* = 3) and SCM (*n* = 7). Clinical data was collated including age of onset, clinical phenotype, genetic mutation and use of medication (Table 1).

For comparison, we reviewed scans of 19 healthy volunteers with similar age range to the patients (12 female, 7 male, mean age 32 years, range 21–55 years) who had been imaged using the same sequences as part of a parallel research study with approval of the local ethics committee.

### 2.2 MRI protocol

All subjects were scanned at 3T (Siemens TIM Trio, Erlangen, Germany) in a supine position with surface array coils to receive the signal from the thighs and calves of both limbs. Patients were scanned with a clinical imaging protocol comprising T1w axial imaging (representative parameters: repetition time (TR)/echo time (TE) = 575/19 ms, number of signal averages (NEX) = 1, 384 × 228 matrix) and axial STIR imaging (representative parameters TR/TE/inversion time (TI) = 4900/47/220 ms, 2 signal averages, 320 × 192 matrix) with 5 mm slice thickness and 1 mm slice gap. Sequence parameters varied between individuals to ensure full anatomical coverage and to meet specific absorption rate restrictions, with scanning time being usually less than 20 min. Healthy volunteer images were drawn from an existing study dataset with T1w: TR/TE = 671/16 ms, NEX = 1 and STIR: TR/TE/TI = 5500/56/220 ms, NEX = 1, both with 10 mm slice thickness, 10 mm slice gap and a 256 × 192matrix.

### 2.3 MRI analysis

We first analyzed individual muscles and subsequently categorized the overall degree of involvement at thigh and calf level. The following individual muscles were assessed bilaterally: rectus femoris, vastus intermedius, vastus lateralis, vastus medialis, semimembranosus, semitendinosus, biceps femoris, adductor magnus, gracilis and sartorius in the thigh; tibialis anterior, peroneus longus, medial gastrocnemius, lateral gastrocnemius, soleus and tibialis posterior in the calf. These 32 muscles were assessed on T1w sequences for the presence of fatty infiltration using Fischer’s semi-quantitative scale [8]: 0 – normal appearance, 1 – occasional scattered T1 hyperintensity, 2 – confluent areas of T1 hyperintensity <50% of muscle involved, 3 – confluent areas of T1 hyperintensity >50%, 4 – complete replacement of muscle with fat. The same muscles were assessed on the STIR sequences for presence of hyperintensity indicative of oedema and were graded on a three point scale: 0 – absent, 1 – mild or 2 – definite hyperintensity. Patient MRI scans were independently analysed by two neuroradiologists with post-specialist experience in neuromuscular imaging (three years: author AF, one year: IA in acknowledgements) who were blinded to clinical details. There was exact agreement in Fischer grading for 81% of muscles, whilst 17% of muscles differed by a single grade. All disagreements in ratings were resolved in a consensus meeting of these two radiologists together with a senior neuroradiologist with over 20 years post-speciality experience (author TY). In a separate session a single neuroradiologist (AF) analyzed the scans from the 19 healthy volunteers using the same methods.

Taking into account the volunteer data, we created an overall categorization for each sequence. The T1 weighted sequences (thighs or calves) were categorized as:

• Normal: all muscles grade 0.
• Mild limited changes: grade 1 changes in ⩽50% of the muscles (⩽10/20 in thighs; ⩽6/12 in calves).
• Mild extensive changes: grade 1 changes in >50% of the muscles.
• Marked changes: any muscle with grade 2 changes.

The STIR sequences were similarly categorized:

• Normal: all muscles normal intensity.
• Mild limited changes: mild hyperintensity in ⩽1/3 of the muscles (⩽6/20 in thighs; ⩽4/12 in calves).
• Mild extensive changes: mild hyperintensity in >1/3 of the muscles.
• Marked changes: any muscle with marked hyperintensity.

### 2.4 Statistical considerations

For the purposes of pattern analysis left and right values for each patient were averaged and only descriptive statistics were used. An overall MRI involvement score was established for each patient by calculating the mean of the qualitative scores of all 32 muscles. Spearman’s rank correlation coefficient was used to assess correlation between overall MRI involvement score and age. Statistical analyses were performed using PASW Statistics 18, Release Version 18.0.0 (= D3 SPSS, Inc., 2009, Chicago, IL, www.spss.com).

## 3 Results

**Fig. 1:**
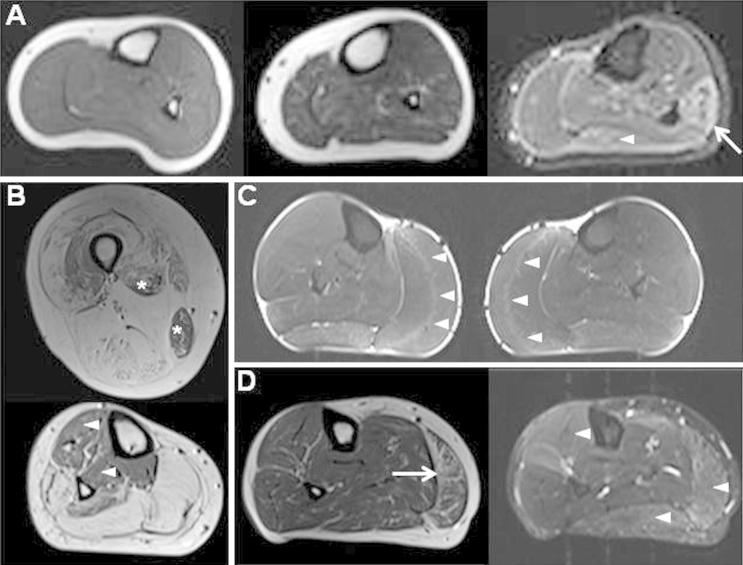
(A) Range of appearances in healthy volunteers. Left (T1w right calf): normal appearance of all muscles; middle (T1w right calf): mild streaks of hyperintensity in some muscles, right: (STIR left calf) mild STIR hyperintensity in lateral gastrocnemius (arrowhead), marked STIR hyperintensity in peroneus longus (arrow). (B) 43 year old man with SCM (patient 17). Top (T1w right thigh): severe fatty changes with almost complete replacement of the hamstrings by fat, with relative sparing of the adductor longus and gracilis (asterisks); bottom (T1w right calf): complete fatty replacement of the posterior superficial compartment with sparing of tibialis anterior and tibialis posterior (arrowheads). (C) 40 year old man with dominant MC (patient 1), STIR both calves. The “central stripe” in both left and right medial gastrocnemius muscles (arrowheads) and mild hyperintensity in both lateral gastrocnemius muscles. (D) 58 year old woman with PMC (Patient 13). Left (T1w right calf) Marked T1 changes with confluence of T1 hyperintensity evident in medial gastrocnemius (arrow); right (STIR right calf): STIR hyperintensity in many calf muscles (arrowheads).

The MRI examinations of all 21 patients showed hyperintensity within muscles on either T1w or STIR images. The categorization of T1w and STIR, thigh and calf images is detailed in Table 1 (patients) and Table 2 (volunteers) and is summarized by group in Table 3. Representative images from patients and volunteers are shown in Fig. 1. Further images are available in online Supplementary material, Fig. e1.

### 3.1 T1w abnormalities

**Fig. 2:**
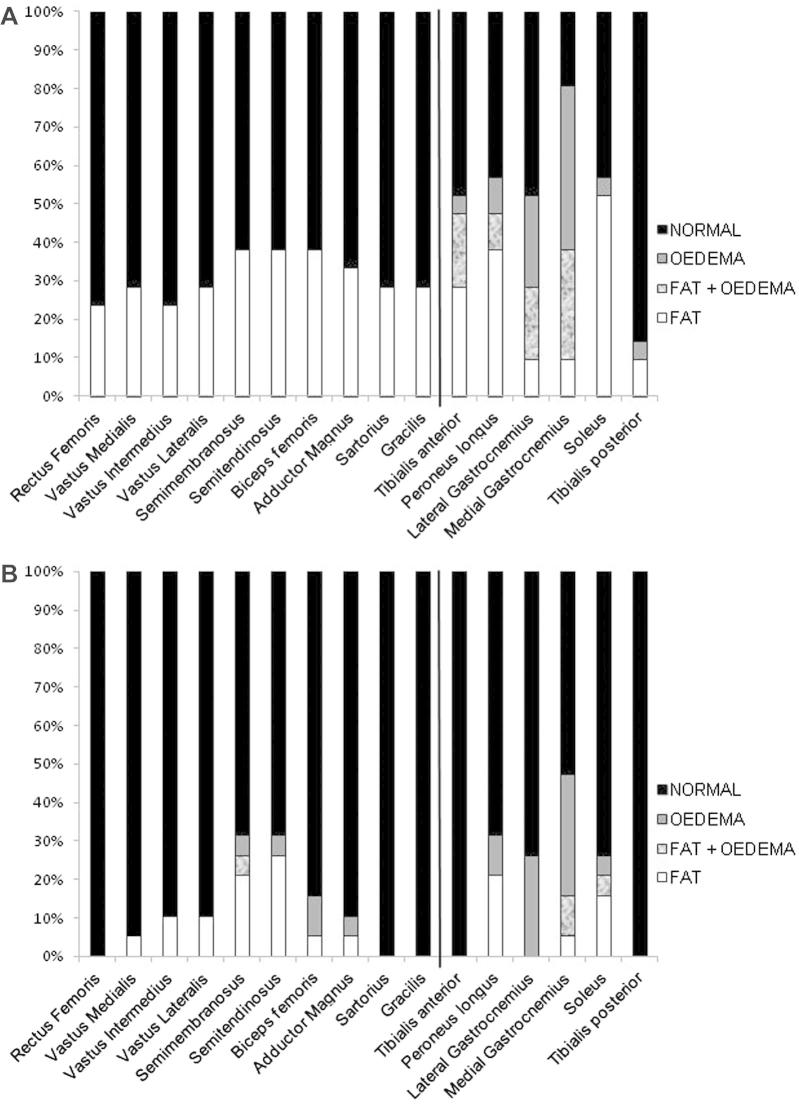
Distribution of fatty infiltration and oedematous changes across thigh and calf muscles. (A) Patients, (B) healthy volunteers.

All scans were adequate in quality and coverage to allow assessment of all defined muscles in all subjects, Fatty infiltration was found in 30% (128/420) of thigh muscles and in 37% (94/252) of calf muscles in patients, and in 8% (32/380) of thigh muscles and 9% (21/228) of calf muscles in volunteers. In volunteers all changes were Fischer grade 1. In patients, the fatty infiltration covered all grades: thigh: grade 1: 101 muscles (between 7 patients), grade 2: 9 muscles (4 patients), grade 3: 8 muscles (1 patient), grade 4: 10 muscles (1 patient); calf: grade 1: 78 muscles (14 patients), grade 2: 7 muscles (4 patients), grade 3: 5 muscles (1 patient), grade 4: 4 muscles (1 patient). All grade 3 and 4 muscles were from a single patient (patient 17 in Table 1, see also Fig. 1B). Within the limitations of small group sizes there did not appear to be differences between clinical subgroups in distribution of muscles involved within the calf or thigh. There was not a specific pattern of muscle involvement in the thigh (Fig. 2A left), although one patient had markedly different degrees of involvement between muscles (Fig. 1B). In the calf however, there was relative sparing of fatty changes in tibialis posterior (2/21) (Fig. 2A right).

Overall fatty changes in patients were categorized as mild extensive in 14% (3/21) and marked in 19% (4/21), for both calf and thigh sequences (Tables 1 and 3). While a proportion of both patient and control T1w thigh or calf images were categorized as having mild limited changes, no volunteer scans were assigned to higher categories (Tables 2 and 3).

### 3.2 STIR abnormalities

STIR hyperintensity, reflecting oedema, was not identified in any of the thigh images, but was identified in the calf images in 90% (19/21) of patients. Mild STIR hyperintensity in calf muscles was found in 53% (10/19) of healthy volunteers but was usually limited to the gastrocnemius muscles and was categorized as mild limited changes in eight of the volunteers.

The STIR hyperintensity in patients was more commonly categorized as mild extensive or marked than in volunteers (8/21 vs 2/19). In patients STIR hyperintensity was most common in the medial gastrocnemius muscle (15/21), followed by the lateral gastrocnemius muscle (9/21 patients) and tibialis anterior muscle (5/21 patients) (Fig. 2A). The STIR hyperintensity in calf muscles seen in patients was more widespread and of greater intensity than in healthy volunteers (Fig. 2B).

During the analysis, we noticed the presence of a hyperintense stripe in the medial gastrocnemius in a number of patients (Fig. 1C). We therefore assessed the frequency of this finding in a separate session. The presence of his feature was defined as a hyperintense stripe identifiable on more than one slice, parallel to the external circumference of the muscle and extending more than one third of the muscle on an axial image. The central stripe was present in 14/21 patients (10/11 CLCN1, 3/10 SCNA4). The central stripe was not present on any of the scans of the 19 healthy volunteers. The central stripe was present in 75% (6/8) of patients whose scans otherwise showed only mild limited changes (Table 1).

### 3.3 Clinical MRI correlations

**Fig. 3:**
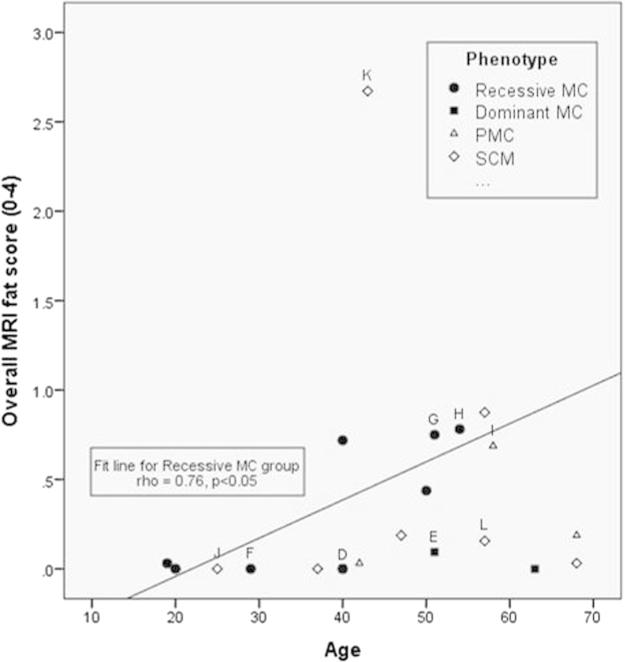
Correlation between age and fatty infiltration on MRI. Scatter-plot of age versus fat score in all 21 patients. (A) significant correlation (rho = 0.46, *p* < 0.05) is observed, which is stronger in the largest subgroup, the recessive myotonia congenita group (rho = 0.76, *p* < 0.05, trend line shown). Marked points refer to sample images in Fig. e1. MC: myotonia congenita, PMC: paramyotonia congenital, SCM: sodium channel myotonia.

The correlation between overall MRI involvement and age was significant (rho = 0.46, *p* < 0.05) despite the diversity in phenotype and genotype. The correlation was stronger (rho = 0.76, *p* < 0.05) for the largest subgroup, recessive MC (Fig. 3). The average number of muscles with oedema was similar in patients on treatment (*n* = 14, 3.4) and patients not on treatment (*n* = 7, 3.3).

### 3.4 Overall spectrum of imaging findings

There was a wide spectrum of findings when all images were considered together for each patient (Table 1, Fig. 1). A total of 10/21 patients had at least mild extensive T1w changes compared with no volunteers. One patient showed severe fatty replacement of some muscles and marked variation between muscles but no STIR hyperintensity. Five further patients had marked T1w changes (Fischer grade 2) in some muscles with additional STIR abnormality in four of these. A further 4 patients had mild extensive T1w changes in muscles of the thigh, calf or both with additional STIR abnormalities in all.

Of the 11 patients without definite T1w changes, 9 had the central stripe either with (3 patients) or without (6 patients) additional mild extensive STIR hyperintensity in the calf. This leaves just two patients who had only mild limited abnormalities and lay within the spectrum of healthy volunteer findings.

## 4 Discussion

We have described the spectrum of MRI abnormalities in genetically confirmed non-dystrophic myotonia. We identified signal abnormalities in all patients. Almost half (10/21) of the patients had at least mild extensive T1w changes which were not found in any healthy volunteers. Furthermore a “central stripe” of STIR hyperintensity was found in 13/21 patients and in none of the healthy volunteers. Only two patients had changes categorized as no more than mild limited.

Very few studies have examined MRI changes in NDM. One study [11] assessed sodium MRI spectroscopy in patients with SCN4A gene mutations. The imaging protocol included T1w, T2w and STIR sequences. They reported T2w abnormalities in one of sixteen NDM patients overall although T2w abnormalities were more common following cooling in the PMC group. The T1w and STIR results were not specifically reported. A further study [12] reported no abnormalities in three patients with recessive myotonia congenita using whole body MRI including T1w, T2w and fat-suppressed T2w sequences. Differences in age could be a contributing factor to this discrepancy. In our study there were less T1w changes in young compared to older recessive MC were less in young patients than older patients (see Fig. 3, filled circles). Subtle abnormalities such as the central stripe of STIR hyperintensity may be more apparent in our series due to the use of dedicated lower limb rather than whole body protocols [12].

Although collectively termed “non-dystrophic” myotonias to differentiate them from myotonic dystrophy, the finding of abnormalities on MRI in patients with myotonia congenita, sodium channel myotonia and paramyotonia congenita is not unexpected. Imaging abnormalities are in keeping with the clinical observation that a proportion of these patients develop fixed weakness and muscle biopsy findings in these patients may show myopathic changes, tubular aggregates and vacuolar changes [3]. Our results are consistent with an ultrasound study of NDM patients which showed increased echogenicity suggestive of fatty infiltration or fibrosis of left biceps brachii, right forearm flexors and left tibialis anterior compared with healthy volunteers [13]. However, ultrasound examination is limited to superficial muscle groups and is operator dependent with relatively low inter-observer and intra-observer agreement [9].

### 4.1 T1w MRI pattern

Although the degree of fatty infiltration was mild overall, the group was heterogeneous ranging from normal T1w scans (7/21 patients) to a patient with severe changes (Table 1). No specific pattern within thigh or calf muscles distinguished clinical subgroups of patients. The diffuse pattern of fatty infiltration noted in this study is different from that reported in patients with myotonic dystrophy where more selective involvement of muscles is seen especially in myotonic dystrophy type 1 [14]. This may help differentiate NDM from other myotonic disorders.

The degree of abnormality on T1w MRI is greater than that seen in healthy volunteers in whom T1 signal hyperintensity was noted in 9% of individual muscles in this study. Overall 8/19 volunteers were categorized as having mild limited T1w changes at thigh or calf level. That scattered area of T1 hyperintensity within muscle may be seen in healthy adult volunteers is an important observation of this study. A recent publication included semi-quantitative assessment using a scale broadly similar to the Fischer scale of T1w calf MRI in children with Duchenne muscular dystrophy and healthy children. The mean grade assigned to different calf muscles in healthy children ranged between 0.5 and 1.1 [15]. The mean grade in our adult healthy volunteers at calf level ranged between 0 and 0.2. Although the mean age and gender distribution differed slightly between the patient group and control group, there was no evidence in this study that average Fischer grading increased with age in the healthy volunteers (rho = 0.12, *p* = 0.63), nor any gender difference (*p* = 0.65). There were also minor differences in acquisition parameters such as slice thickness in some subjects, however these are unlikely to have a significant effect on qualitative Fischer grading.

This study stresses the importance of assessing the spectrum of MRI appearances in volunteers, to allow better categorisation of the relevance of mild changes in patients. In this study the cut-off of any muscle with confluent T1w hyperintensity (Fischer grade 2) or more than half muscles with streaks of hyperintensity (Fischer grade 1) was effective in separating the patient and volunteer groups and might be used as a benchmark for future studies. However, if the detection of mild abnormalities in a group of patients is required, a suitably matched control group should be considered as these abnormalities may vary depending on age or gender.

### 4.2 STIR MRI pattern

STIR hyperintense changes were muscle specific. No STIR hyperintensity was detected in the thigh muscles of any of the patients. However, STIR abnormalities (the central stripe, mild extensive or marked hyperintensity) were detected in the calves of 90% of patients and 10% of volunteers. The most commonly involved muscle was the medial gastrocnemius followed by the lateral gastrocnemius and the tibialis anterior muscles. The pattern of hyperintensity within the muscle was striking with a central stripe of STIR hyperintensity in the medial gastrocnemius of 10 out of 11 patients with myotonia congenita and 3 out of 10 patients with PMC/SCM. We did not see a similar pattern in our healthy volunteers and to our knowledge, it has not been reported to date in any other diseases. The central stripe could be an MRI sign suggestive of non-dystrophic myotonia, especially myotonia congenita and could therefore be an additional aid to guiding genetic testing.

The specific localization of the STIR hyperintensity as a central stripe within the medial gastrocnemius is of interest. Anatomically the central stripe corresponds to the motor endplate zone of medial gastrocnemius due to the pinnate arrangement of muscle fibers which pass from superficial to deep as they run from proximal to distal [16]. Furthermore animal studies have shown greatest concentration of CLCN1 channels at the neuromuscular junction [17], which may explain the increased susceptibility of this region to pathological changes in patients with myotonia congenita.

Hyperintensity on STIR imaging is commonly referred to as “muscle oedema”; although the exact correlate of this term at the tissue level is poorly understood. At a basic level STIR hyperintensity results from prolongation of T2 relaxation times due to changes in tissue water distribution. Increased T2 times have been documented in healthy muscle after exercise due to a number of proposed mechanisms including water shift from intra- to extra-cellular spaces, increase in extra-cellular or vascular fluid volumes, and increase in proportion of “free” water to macromolecular “bound” water [18]. Considering that myotonia represents abnormally prolonged muscle contraction, similar mechanisms may be the cause of STIR hyperintensity in NDM. Further insight is gained from a sodium spectroscopy MRI study which included patients with SCM and PMC. They found reduced intracellular sodium concentration at baseline which increased following cold provocation. All PMC patients also developed STIR hyperintensity after provocation postulated to be due to the accumulation of water within muscle fibers driven by the increased intracellular sodium concentration [11].

We detected evidence of abnormal muscle water distribution in 90% of NDM patients without specific provocation and only a small minority (2/19) of healthy volunteers using the cut-off for abnormal of >1/3 of muscles mildly hyperintense, or at least one muscle with marked STIR hyperintensity, or the central stripe of hyperintensity within the medial gastrocnemius muscle. This cut-off definition was useful to divide the mild STIR hyperintensity seen in a small number of muscles in some volunteers from abnormal STIR hyperintensity within the patient group.

**Supplementary Fig. 1:**
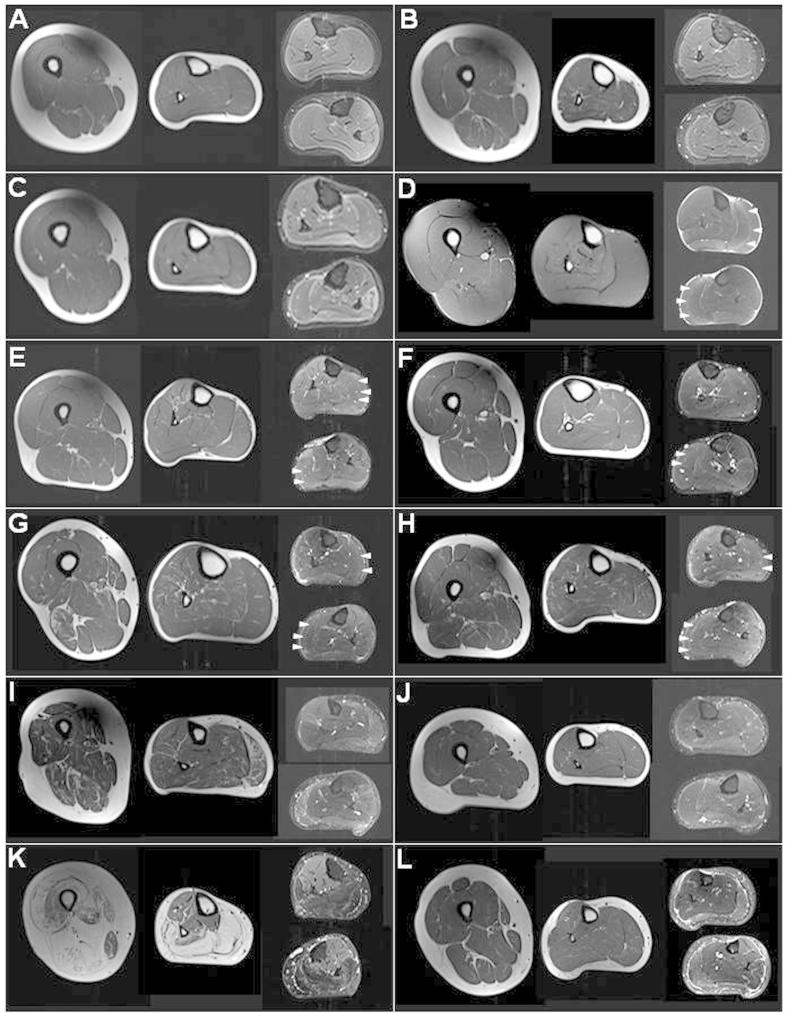


This STIR hyperintensity in volunteers has not to our knowledge been previously reported, though qualitative analysis of skeletal muscle MRI in healthy volunteers is rare [15]. The hyperintensity was mild in degree and generally in just the distal portion of medial or lateral gastrocnemius. The typical appearance is depicted in lateral gastrocnemius bilaterally in Supplemental Fig. 1C. This was overall seen in 8/19 volunteers in medial gastrocnemius and 5/19 volunteers in lateral gastrocnemius, and did not appear to be due to field inhomogeneity or inadequate fat suppression. Quantitative studies have reported longer T2 in medial gastrocnemius than tibialis anterior in healthy volunteers [19,20] or the variation may represent differences in recent exercise which can also affect muscle T2 [21] but wasn’t specifically controlled for in this qualitative study. The STIR findings again highlight the importance of comparison with healthy volunteers to prevent misclassification of mild hyperintensity in some muscles as abnormal. Future studies could investigate this in greater detail by using a more sophisticated scale to describe STIR changes, such as that proposed by Poliachik [22] or by applying quantitative methods to measure muscle T2.

### 4.3 Clinical implications

There was correlation between age and overall MRI score in the overall patient group. This correlation was even stronger in the recessive MC subgroup suggesting that T1w MRI changes progress with age however a longitudinal study would be needed to confirm this. Longitudinal imaging would also answer important questions regarding the temporal relationship between oedema and fatty infiltration. Although no correlation was seen between medication use and number of muscles with STIR hyperintensity, this might be explained by the worse affected patients being more likely to be on medications. Whether sequences sensitive to changes in water distribution might be able to detect a treatment affect will need to be assessed in an interventional or longitudinal study.

The fatty infiltration of muscles seen in this series clearly indicates that progressive irreversible muscle damage does occur in some patients with non-dystrophic myotonia. It is unknown if any of the current therapies used in NDM have any influence on the development of myopathy and it is not known whether medication can reverse the MRI abnormalities in this group of patients, although such a reversal has been demonstrated in hypokalaemic periodic paralysis [10]. By combining methods to quantify abnormal water distribution [10] with muscle fat measurements [23], MRI may also be useful as an outcome measure in future treatment trials of non-dystrophic myotonia.

In conclusion, lower limb muscle MRI in patients with non-dystrophic myotonia revealed fatty infiltration and oedema in the majority of patients. Although T1w changes in most patients were mild overall, hyperintensity was present to a greater degree than in controls and some patients demonstrated extensive fatty infiltration. STIR changes were also more common and furthermore, we described a previously unreported STIR hyperintense central stripe within the medial gastrocnemius muscle, which we identified consistently especially in patients with myotonia congenita and could be of diagnostic use.

## Figures and Tables

**Table 1:** Clinical characteristics of patients and MRI findings.

ID	Age	Sex	Channel	Mutation	Phenotype	Medication	Thigh		Calf			Overall score
							T1	STIR	T1	STIR	Stripe	
1	40	M	CLCN1	Gly285Glu	Dominant MC	Nil	−	−	−	+	Yes	0
2	51	F	CLCN1	Ala313Val	Dominant MC	Disopyramide	−	−	++	++	Yes	0.13
3	63	M	CLCN1	Gly230Glu	Dominant MC	Mexiletine	−	−	−	++	Yes	0
4	19	M	CLCN1	c.[1261C>T (+) 2596–11C>G]; p.[Arg421Cys (+) ?]	Recessive MC	Mexiletine	−	−	±	±	Yes	0.04
5	20	F	CLCN1	c.[180+3A>T (+)1251+4A>C]; p.[? (+) ?]	Recessive MC	Nil	−	−	−	−	Yes	0
6	29	M	CLCN1	p.[Gly222Ser;Arg976X]+[Pro558Ser]	Recessive MC	Mexiletine	−	−	−	−	Yes	0
7	40	M	CLCN1	c.[696+2_696+10del(+)1183_1187del]; p.[?(+)Gly395fs]	Recessive MC	Mexiletine	+	−	±	++	No	0.63
8	40	M	CLCN1	c.1167–10T>C & deletion of exons 8–10	Recessive MC	Mexiletine	−	−	−	++	Yes	0
9	50	M	CLCN1	c.[180+3A>T (+) 568G>A]; p. [? (+) Gly190Arg]	Recessive MC	Mexiletine	+	−	±	−	Yes	0.40
10	51	M	CLCN1	p.[Val327Ile (+) Arg894X]	Recessive MC	Quinine	++	−	±	±	Yes	0.65
11	54	F	CLCN1	c.[180+3A>T (+) 568G>A]; p. [? (+) Gly190Arg]	Recessive MC	Mexiletine	+	−	+	±	Yes	0.71
12	42	F	SCN4A	Thr1313Met	PMC	Nil	−	−	±	±	Yes	0.04
13	58	F	SCN4A	Arg1448Leu	PMC	Phenytoin	++	−	++	+	No	0.65
14	68	M	SCN4A	Thr1313Met	PMC	Mexiletine	−	−	++	++	No	0.25
15	25	F	SCN4A	Gly1306Ala	SCM	Mexiletine	−	−	−	±	No	0
16	37	F	SCN4A	Gly1306Ala	SCM	Nil	−	−	−	±	Yes	0
17	43	M	SCN4A	Leu1436Pro	SCM	Nil	++	−	++	−	No	2.43
18	47	M	SCN4A	Val1589Met	SCM	Nil	±	−	±	±	Yes	0.17
19	57	F	SCN4A	Val1589Met	SCM	Nil	−	−	+	++	No	0.21
20	57	F	SCN4A	Leu128Pro	SCM	Mexiletine	++	–	+	±	No	0.80
21	68	M	SCN4A	Gly1306Ala	SCM	Mexiletine	−	−	±	−	No	0.04

M: male, F: female, CLCN1: voltage-sensitive chloride channel gene, SCN4A: voltage-gated sodium channel gene, MC: myotonia congenita, PMC: paramyotonia congenita, SCM: sodium channel myotonia, STIR: short-tau-inversion-recovery. −: all muscle normal, ±: mild limited changes, +: mild extensive changes, ++: marked changes (as defined in the methods). Overall score: mean T1 grade across all muscles.

**Table 2:** Summary of MRI findings on thigh and calf imaging in healthy volunteers.

Number of volunteers	Thigh		Calf			Overall score
	T1	STIR	T1	STIR	Stripe	
8	−	−	−	−	No	0
1	−	−	−	±	No	0
2	−	−	±	±	No	0.08
2	±	−	−	±	No	0.08
1	−	±	−	±	No	0
2	±	−	±	±	No	0.34
1	±	±	±	−	No	0.47
1	−	±	−	++	No	0
1	±	−	±	+	No	0.19

The volunteers are grouped according to scan findings. − = all muscle normal, ± = mild limited changes, + = mild extensive changes, ++ = marked changes (as defined in the methods). Overall score: mean T1 grade across all muscles.

**Table 3:** Overall categorization of T1w and STIR sequences in patients and controls.

Sequence	No abnormalities	Mild limited	Mild extensive	Marked
*T1 weighted sequences*				
Thigh patient	62% (13/21)	5% (1/21)	14% (3/21)	19% (4/21)
Thigh control	68% (13/19)	32% (6/19)	0	0
Calf patient	33% (7/21)	33% (7/21)	14% (3/21)	19% (4/21)
Calf control	68% (13/19)	32% (6/19)	0	0

*STIR sequences*				
Thigh patient	100% (21/21)	0	0	0
Thigh control	84% (16/19)	16% (3/19)	0	0
Calf patient	24% (5/21)	38% (8/21)	10% (2/21)	29% (6/21)
Calf control	47% (9/19)	42% (8/19)	5% (1/19)	5% (1/19)

See methods for category definitions.
